# Impact of neoadjuvant chemotherapy on thrombus viability in patients with Wilms tumour and caval extension: systematic review with meta-analysis

**DOI:** 10.1093/bjsopen/zrab020

**Published:** 2021-05-30

**Authors:** T D Boam, M Gabriel, R Shukla, P D Losty

**Affiliations:** Department of Paediatric Surgery, Chelsea and Westminster Hospital, London, UK; Department of Paediatric Surgery, Norfolk and Norwich Hospital, Norwich, UK; Department of Pathology, Alder Hey Children’s Hospital, Liverpool, UK; Alder Hey Children’s Hospital NHS Foundation Trust, School of Health and Life Science, University of Liverpool, UK

## Abstract

**Background:**

Inferior vena cava (IVC) tumour thrombus in children with Wilms tumour is typically managed with neoadjuvant chemotherapy with the intention of achieving thrombus regression in order to minimize the risks associated with complex vascular surgery.

**Methods:**

A systematic review of Medline and Embase databases was undertaken to identify all eligible studies with reference to thrombus viability in Wilms tumour index cases with caval/cardiac extension. A meta-analysis of proportions was utilized for pooled thrombus viability data across studies. Logistic regression was used to analyse the relationship between thrombus viability and duration of chemotherapy.

**Results:**

Thirty-five eligible observational studies and case reports met inclusion criteria describing a total of 236 patients with thrombus viability data. The pooled proportion of patients with viable tumour thrombus after neoadjuvant chemotherapy was 0.53 (0.43–0.63). Logistic regression analysis of 54 patients receiving either a standard (4–6 weeks) or extended (more than 6 weeks) course of neoadjuvant chemotherapy resulted in an odds ratio of 3.14 (95 per cent c.i. 0.97 to 10.16), *P* = 0.056, with extended course therapy trending towards viable tumour thrombus.

**Conclusion:**

Preoperative chemotherapy is successful in achieving non-viability of caval and cardiac thrombi in around 50 per cent of children, without added benefit from extended cycles of neoadjuvant chemotherapy. Risks *versus* benefits of extirpative vascular surgery must be considered, therefore, for these high-risk patients.

## Introduction

Intravascular thrombus extension is a recognized hallmark of Wilms tumour, with extension into the inferior vena cava (IVC) in 4–10 per cent of cases^1^ and intracardiac lesions observed in 1–3 per cent of cases^2–4^. The thrombus in most cases is neoplastic, containing malignant cells disseminated from primary tumour growth. The Daum staging system classifies disease based on its level above or below the hepatic veins, intimal vessel involvement and whether there is right atrial or ventricular tumour extension, to aid surgeons in planning successful resection^3^^,^^5^. Patients with supradiaphragmatic extension often require cardiopulmonary bypass (CPB) or deep hypothermic circulatory arrest to extract thrombi^1^^,^^3^^,^^4^^,^^6^. For others, proximal and distal IVC occlusion alone may be sufficient^1^^,^^7–9^. Both the National Wilms Tumour Study (NWTS) Group and The International Society of Paediatric Oncology (SIOP) protocols advocate preoperative chemotherapy to reduce perioperative complications and induce thrombus regression prior to radical nephrectomy to obviate the need for direct caval surgery or CPB^1^^,^^4^^,^^10–12^.

Thrombectomy is undertaken for oncological control to achieve a complete resection, but may be required urgently to prevent complications from its haemodynamic effects, including tumour embolus and hepatic and cardiac failure^13–16^.

Mobile tumour thrombi are cleared by cavotomy and thrombectomy, but when thrombus is densely adherent to the vein wall, intimal dissection may be needed for total ‘piecemeal’ extraction^8^^,^^17^^,^^18^. This risks caval narrowing and secondary thrombotic occlusion^1^^,^^6^^,^^17^. Where thrombectomy is not feasible or the IVC is totally occluded by thrombus, partial or full cavectomy may be tolerated due to collateral venous flow^3^^,^^4^^,^^19–21^. Caval repair can be undertaken by direct suturing or with bovine or autologous pericardial patches or synthetic grafts as necessary^4^^,^^15^^,^^20^. Cavectomy carries significant added risk to the patient, relating to the increased complexity of the operation; potential inadequate collaterals with venous pooling in lower extremities, refractory ascites and direct hazards to the contralateral kidney^17^. Some, but not all, patients require caval reconstruction, depending on their haemodynamic response when the IVC is clamped at the suprahepatic level, reflecting the adequacy of the collateral circulation^17^. When the left kidney is left *in situ*, rich collaterals may develop from the gonadal, adrenal, ascending phrenic and lumbar veins as well as the azygos system^19^^,^^22^. Where the right kidney is left behind, a renoportal shunt may be required. Massive haemorrhage and death are well recognized, and likely under-reported, complications of caval and cardiac surgery^4^^,^^9^^,^^20^.

Wilms tumour patients with caval or cardiac extension (CCE) have comparable survival outcomes to those without vascular invasion and, although results of the third and fourth National Wilms Tumour Study (NWTS) showed the incidence of overall surgical complications to be decreasing, children with CCE are still categorized as a high-risk group^1^^,^^23–25^. It remains controversial whether removal of tumour thrombus is necessary in those patients where doing so would pose a significant risk to life. Preoperative chemotherapy and adjuvant postoperative radiotherapy may conceivably be sufficient to achieve oncological control of malignant thrombus.

This study therefore investigated the effect of neoadjuvant chemotherapy on thrombus viability, in order to determine if complete thrombectomy is essential to achieve macro- and microscopically clear resection margins.

## Methods

### Systematic review

A systematic review was undertaken in accordance with the Preferred Reporting Items for Systematic reviews and Meta-Analysis (PRISMA) guidelines^26^. Medline and Embase (Ovid^®^) databases were searched using the terms: Wilms, nephroblastoma, caval, vena cava, IVC, thrombus, intra-atrial, intracaval, intracardiac, intravascular, vascular, atrial, and atrium. Limits were set at human subjects, English language publications, patient ages 0–18 years and studies from 1990 onwards. Searches were undertaken in April and May 2020. Title and/or abstract screening was undertaken independently by two study authors to identify original case reports, observational series and randomized trials including Wilms tumour with intravascular extension. Full texts were then retrieved and searched by a single author for references to paediatric Wilms tumour with intravascular extension beyond the renal vein, which received neoadjuvant chemotherapy, then nephrectomy and thrombectomy with subsequent full histological analysis of thrombus. Patients who did not receive neoadjuvant chemotherapy or who did not require thrombectomy were excluded. Eligible conference abstracts were also included. Where information about chemotherapy or thrombus histology was incomplete, authors of these studies were contacted directly by email. Tumour thrombi were classified as ‘viable’ if any active tumour cells were reported on histology examination. Reports of complete necrosis of the thrombus with no active tumour cells were classified as ‘non-viable’. Consensus agreement between all three study authors was used where data reporting was unclear. The most recent study was selected where there were multiple reports from the same institution. Reference lists of all included studies were manually searched.

### Statistical analysis

StatsDirect software (StatsDirect Ltd Liverpool, UK) was used. A meta-analysis of proportions analysed pooled thrombus viability data across studies and a random effects model was applied. Logistic regression was used to analyse the relationship between thrombus viability and duration of chemotherapy, using data from individual patients where complete and amenable to comparison. Patients were then categorized by the duration of neoadjuvant chemotherapy as: short course, less than 4 weeks; standard course, 4–6 weeks; or extended course, more than 6 weeks^27^. Where the duration of neoadjuvant chemotherapy was reported in terms of cycles, courses, doses or protocols, standardized Wilms tumour regimens for the same agents were used to convert these terms to weeks, for adequate comparisons to be made.

## Results

### Systematic review

**Fig. 1:**
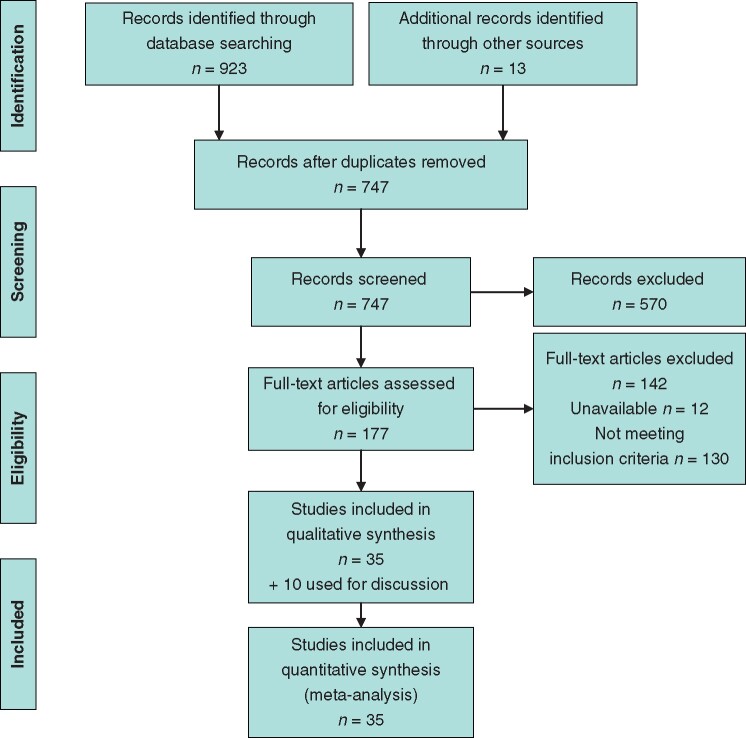
PRISMA flow diagram

The initial search strategy yielded 734 studies after removal of duplicates (*Fig. 1*). After title and abstract screening, 177 articles were retrieved, 36 of which were conference abstracts with 13 studies identified through manual bibliography searching. Sixty-seven authors were also directly contacted with 30 further responses. Twelve articles were unavailable. This resulted in the identification of a total of 35 eligible studies, including five conference abstracts that met the final inclusion criteria. Of these, 20 studies required direct correspondence with study authors to verify and update information (*Table 1*). All included studies on the theme topic were observational, the majority were case reports or small case series, with only four study series involving more than 20 patients. There were no prospective trials comparing interventions for CCE in Wilms tumour.

**Table 1:** Details of all studies

Study	Date	Number of patients receiving preoperative chemotherapy	Number with viable tumour thrombus on resection	Duration of chemotherapy (weeks)	Chemotherapy regimen	Notes
**Elayadi *et al*.** ^25^	2020	31	20	6–12	VCR/ActD/doxorubicin	
**Viswanathan *et al*.** ^13^	2019	1	1	1	VCR/ActD	
**Altwaeel *et al*.** ^28^ *	2019	2	2	Not specified	Not specified	
**Imle *et al*.** ^14^ *	2019	1	0	4	VCR/ActD	
**Tekin *et al*.** ^29^ *	2019	1	0	6	Ifosfamide/carboplatin/etoposide	
**John *et al*.** ^30^ *	2018	1	0	6	VCR/ActD/doxorubicin	
**Sekhon and Suryavanshi** ^31^ *	2018	1	0	6	VCR/ActD	
**Tan *et al*.** ^32^ *	2018	2	0	Not specified	VCR/ActD/doxorubicin	
**Dong *et al*.** ^33^ *†	2018	7	0	4–7	Ifosfamide/etoposide/VCR/ActD/ doxorubicin	
**Cox *et al*.** ^4^ *	2018	11	9	5 (median)	VCR/ActD/doxorubicin	
**Al Diab *et al*.** ^12^	2017	10	8	7 (median)	VCR/ActD/doxorubicin	
**Solomon *et al*.** ^34^ *†	2016	9	4	Not specified	Not specified	
**Bhagat *et al*.** ^35^†	2016	20	13	Not specified	3 drug not specified	Includes 2 patients with renal vein thrombi after chemotherapy
**Genc *et al*.** ^16^ *	2015	1	1	6	VCR/ActD/cyclophosphamide	
**Fawkner-Corbett *et al*.** ^36^ *	2014	6	4	6–16	VCR/ActD +/- doxorubicin	
**Li *et al*.** ^37^ *†	2014	1	0	9 + TACE^*^	Ifosfamide/carboplatin/ etoposide/pirarubicin/ vindesine	Also received transarterial chemoembolization
**Lee *et al*.** ^38^†	2014	3	0	4–9	VCR/ActD/doxorubicin	
**Loh *et al*.** ^17^	2014	10	5	Not specified	Not specified	
**Parelkar *et al*.** ^39^	2013	1	1	8	VCR/ActD/doxorubicin	
**Bader *et al*.** ^8^	2013	9	6	4–6	VCR/ActD/doxorubicin	
**Khozeimeh *et al*.** ^40^	2011	2	2	6, 10	VCR/ActD/doxorubicin	
**Hadley *et al*.** ^41^	2010	31	24	4 (+/- 6 Epirubicin)	VCR/ActD (+/- epirubicin)	
**Cristofani *et al*.** ^42^	2007	9	6	4–6	VCR/ActD	Includes 2 patients with renal vein thrombi after chemotherapy
**Murthi *et al*.** ^15^	2006	11	8	1–29 (10 mean)	VCR/ActD/doxorubicin	Includes 1 primitive neuroectodermal tumour (PNET) and 1 clear cell sarcoma
**Akyüz *et al*.** ^2^	2005	2	0	4	VCR/ActD	
**Szymik-Kantorowicz *et al*.** ^43^	2003	1	0	6	VCR/ActD/epirubicin	
**Renaud *et al*.** ^44^	2001	1	1	10	VCR/ActD/doxorubicin	Patient received neoadjuvant radiotherapy
**Gow *et al*.** ^45^	2001	1	1	Not specified	VCR/ActD/doxorubicin	
**Shamberger *et al*.** ^24^	2001	42	22	8 (median)	VCR/ActD (+/- doxorubicin +/- cyclophosphamide)	5 patients received neoadjuvant radiotherapy
**Giannoulia-Karadana *et al*.** ^46^ *	2000	1	1	6	VCR/ActD/cyclophosphamide	
**Lodge *et al*.** ^7^	2000	1	0	12	VCR/ActD/doxorubicin	
**Sripathi *et al*.** ^47^	2000	1	1	6	VCR/ActD/doxorubicin	
**Matloub** ***et al*.**^48^	1997	1	0	11	VCR/ActD	
**Martìnez-Ibàñez *et al*.** ^49^	1996	1	0	6	VCR/ActD/epirubicin	Intraoperative biopsy from caval thrombus only
**Habib *et al*.** ^50^	1993	3	0	6	VCR/ActD	

* Study required additional unpublished information from authors before inclusion.

† Conference abstract. VCR, vincristine; ActD, actinomycin D.

Thrombus viability data were available for a total of 236 patients with CCE. All patients received preoperative chemotherapy, mostly in line with SIOP and NWTS regimens involving actinomycin D and vincristine with or without doxorubicin.

Eight studies with a total of 86 patients described alternative agents and in two studies preoperative radiotherapy was administered to a total of six patients. In two studies it was not possible to separate histology results fully where the thrombus had regressed to the renal vein, and therefore did not require extensive caval thrombectomy^35^^,^^42^. One study report included two patients with non-Wilms histology^15^, and another publication described the use of transarterial chemoembolization on the primary tumour, in addition to systemic neoadjuvant chemotherapy^37^. These studies were excluded from the sensitivity analysis.

### Meta-analysis

**Fig. 2:**
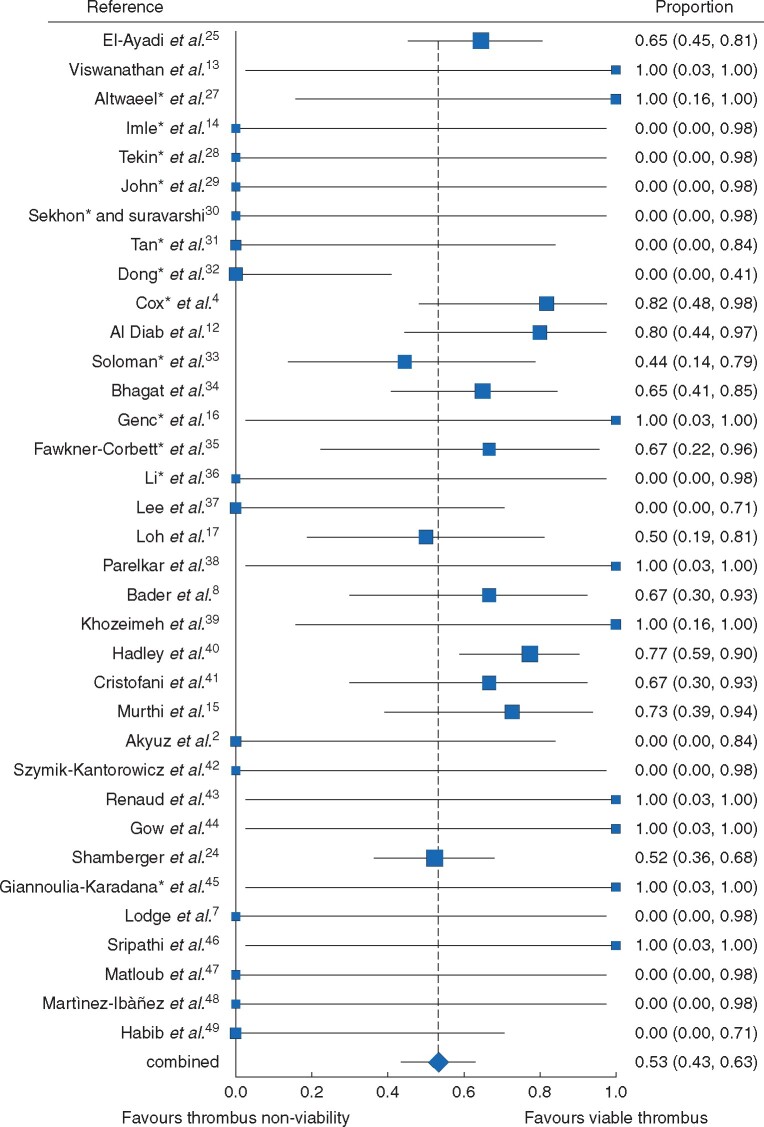
Forest plot – proportion meta-analysis of thrombus viability. ^*^Study required additional unpublished information from authors before inclusion

After qualitative synthesis, it was deemed that the studies were similar enough (*I*^2^ = 46 per cent, moderate heterogeneity) with regards to the outcome of thrombus viability to pool patient data in a meta-analysis. The pooled proportion of patients with viable tumour thrombus after neoadjuvant chemotherapy was 0.53 (0.43–0.63) (*Fig. 2*). A sensitivity analysis, which excluded the five studies that included patients that had neoadjuvant radiotherapy, renal vein thrombi and non-Wilms tumours, yielded similar results to the original analysis, with a proportion of viable tumour thrombus of 0.49 (0.36–0.61) (*Fig.*  *S1*). In the absence of moderate-sized studies, funnel plots indicated significant heterogeneity and asymmetry, suggesting risk of bias (*Fig.*  *S2*).

A total of 54 patients from 21 studies were included in the logistic regression, having had either standard or extended courses of chemotherapy. Short-course neoadjuvant chemotherapy was described in only two patients, both of whom had viable tumour thrombus, so these were therefore excluded. In total, 36 patients received a standard chemotherapy course. Of these, 12 had viable tumour thrombus. The remaining 18 patients received an extended course of chemotherapy with 11 cases having viable tumour thrombus. Logistic regression resulted in an odds ratio of 3.14 (95 per cent c.i. 0.97 to 10.16), *P* = 0.056, trending towards extended courses of chemotherapy having viable tumour thrombus (*Table 2*).

**Table 2:** Logistic regression: standard and extended course chemotherapy as predictors of thrombus viability

Parameter	Odds ratio (95% c.i.)	Z value	*P* (>|Z|)
(intercept)	n/a	−1.960516	0.050
Duration of chemotherapy (two-level dependent variable 0 = standard, 1 = extended course)	3.14 (0.97–10.16)	1.911833	0.056

## Discussion

This study has provided a comprehensive systematic review of thrombus viability after chemotherapy for Wilms tumour using PRISMA methodology. Limitations are acknowledged, reflecting the available literature on this topic. It is highly likely that that the positive outcomes reported by some authors are indicative of publication bias. There are no prospective studies or RCTs specifically focused on surgical or other interventions in management of CCE in Wilms tumour.

The present systematic review did not include a formal assessment of study quality, as there are few validated tools available for the evaluation of case series and case reports and the outcome of interest was specific and not related to overall quality of a study. In addition, a significant number of results were obtained by directly contacting study authors, allowing for an in-depth appraisal of the available literature on this rare condition.

Several common themes were identified related to reporting Wilms tumour with CCE. In several series, the focus was on the thrombus, with the management and outcomes of several types of tumour being described, making it difficult to extrapolate wholly accurate data. Neoadjuvant chemotherapy protocols were often specified but lacked details that might have indicated deviation from regulatory practice. Interpretation of these studies was therefore reliant on direct author contact and comparisons with the standardized regimens. Most studies did not accurately detail radiological or macroscopic tumour response to chemotherapy prior to surgery. It was also not possible to define fully how neoadjuvant radiotherapy influenced thrombus viability or to separate these patients from the series where they were reported, which made exclusion of these studies from the sensitivity analysis necessary. Resection margins were not often discussed, and it remains unclear whether incomplete thrombectomy increases the risk of disease relapse^1^^,^^2^^,^^14^^,^^15^^,^^24^^,^^43^^,^^51^. Adjuvant radiotherapy was usually given in these situations as mandated by tumour-staging protocols, but postoperative chemotherapy, relapse and mortality outcomes were often generalized, and it was difficult to define fully relationships between these and thrombus response and viability. Future studies would benefit from a standardized reporting structure for Wilms tumour patients harbouring CCE, to address these issues and allow greater clarity and comparability of outcomes. This should include a standard system for macroscopic/radiological thrombus response reporting using an existing staging system such as those described by Daum or Hinman^5^^,^^17^.

Meta-analysis was considered appropriate as there was sufficient similarity between the included studies with a binary outcome of interest, whether there was tumour thrombus viability or non-viability. Despite moderate heterogeneity between the included studies, the results of the sensitivity analysis, which excluded studies that described radiotherapy, renal vein thrombi and non-Wilms histology, were similar to those of the main analysis.

A previous meta-analysis found no survival benefits to extended courses of chemotherapy in Wilms patients with intravascular extension, confirmed by the present study^27^. These longer courses of chemotherapy may reflect the lack of radiological regression of thrombus, indicating the absence of macro- or microscopic response, although a single study reported no association between macroscopic appearances and microscopic response^25^. In the present study, no assumptions were made about the comparability of patients given standard or extended courses of chemotherapy. Of the eight studies detailing patients with extended courses, there was no explanation given for the length of treatment, and there were no obvious clinical descriptors to differentiate them from patients who were given standard courses.

The present systematic review raises several key questions and challenges that would be usefully addressed by future prospective studies. Potential areas for investigation include prediction of thrombus viability from novel imaging or biomarkers, efficacy of novel agents for thrombus regression and surgery-led trials comparing clinical outcome metrics with respect to thrombus extraction *versus* chemotherapy and adjuvant targeted radiotherapy.

This systematic review with meta-analysis has confirmed that neoadjuvant chemotherapy was effective in achieving thrombus non-viability in around 50 per cent of patients with tumour extension into the vena cava. This raises the key issue as to whether complex vascular surgery should be considered mandatory for all such patients. Although it is impossible with current imaging technology to determine whether a thrombus is biologically viable or not before surgery, if this could be determined more accurately with innovative tools then the extent of surgery might well be modified in future.

## Supplementary material

Supplementary material is available at *BJS Open* online.

## Supplementary Material

zrab020_Supplementary_Data
