# Supplementary material for: Impact of neoadjuvant chemotherapy on thrombus viability in patients with Wilms tumour and caval extension: systematic review with meta-analysis
Source: BJS Open. 2021 May 30;5(3):zrab020. doi: 10.1093/bjsopen/zrab020 (PMC8164777; doi:10.1093/bjsopen/zrab020)
Supplement: zrab020_Supplementary_Data [file zrab020_supplementary_data.zip › Appendix 2 Funnel plot.docx]

Appendix Figure 2: Funnel plot
